# Dose-Dependent Hemodynamic, Biochemical, and Tissue Oxygen Effects of OC99 following Severe Oxygen Debt Produced by Hemorrhagic Shock in Dogs

**DOI:** 10.1155/2014/864237

**Published:** 2014-10-27

**Authors:** William W. Muir, Carlos L. del Rio, Yukie Ueyama, Bradley L. Youngblood, Robert S. George, Carl W. Rausch, Billy S. H. Lau, Robert L. Hamlin

**Affiliations:** ^1^QTest Labs, 6456 Fiesta Drive, Columbus, OH 43235, USA; ^2^New A Innovation Ltd. 17/F, Chevalier Commercial Centre, 8 Wang Hoi Road, Kowloon Bay, Kowloon, Hong Kong

## Abstract

We determined the dose-dependent effects of OC99, a novel, stabilized hemoglobin-based oxygen-carrier, on hemodynamics, systemic and pulmonary artery pressures, surrogates of tissue oxygen debt (arterial lactate 7.2 ± 0.1 mM/L and arterial base excess −17.9 ± 0.5 mM/L), and tissue oxygen tension (tPO_2_) in a dog model of controlled severe oxygen-debt from hemorrhagic shock. The dose/rate for OC99 was established from a pilot study conducted in six bled dogs. Subsequently twenty-four dogs were randomly assigned to one of four groups (*n* = 6 per group) and administered: 0.0, 0.065, 0.325, or 0.65 g/kg of OC99 combined with 10 mL/kg lactated Ringers solution administered in conjunction with 20 mL/kg Hextend IV over 60 minutes. The administration of 0.325 g/kg and 0.65 g/kg OC99 produced plasma hemoglobin concentrations of 0.63 ± 0.01 and 1.11 ± 0.02 g/dL, respectively, improved systemic hemodynamics, enhanced tPO_2_, and restored lactate and base excess values compared to 0.0 and 0.065 g/kg OC99. The administration of 0.65 g/kg OC99 significantly elevated pulmonary artery pressure. Plasma hemoglobin concentrations of OC99 ranging from 0.3 to 1.1 g/dL, in conjunction with colloid based fluid resuscitation, normalized clinical surrogates of tissue oxygen debt, improved tPO_2_, and avoided clinically relevant increases in pulmonary artery pressure.

## 1. Introduction

Hemorrhage is a major cause of morbidity and mortality in human and veterinary medicine [1, 2]. Multiple animal models have established the pathophysiologic consequences of controlled and uncontrolled hemorrhage, the therapeutic importance of the timely return of normal hemodynamic values, and the restoration of microvascular perfusion [3, 4]. Evidence generated from studies conducted in large animal species (dog, pig, and humans) has identified oxygen debt and tissue oxygen-tension (tPO_2_) values that are predictive of both a therapeutic time window and the fluid replacement volume required to prevent irreversible shock [3, 5, 6].

Oxygen (O_2_) carrying blood substitutes (O_2_ therapeutics) were developed to address concerns regarding the availability, storage, transport, and safety of the human blood supply [7, 8]. Multiple cell-free hemoglobin based O_2_ carriers (HBOCs) and gas-carrying fluids have evolved with most demonstrating the ability to improve hemodynamics, restore tissue oxygen tension, reduce transfusion requirements, and delay definitive care compared to the administration of nonoxygen carrying crystalloids or colloids [9]. Unfortunately, although thoughtfully designed, HBOCs have been fraught with unforeseen dose related adverse effects including vasoconstriction, systemic and pulmonary hypertension, oxidation to methemoglobin, and the possibility of tissue toxicity due to the generation of reactive O_2_ species, effects that have been primarily attributed to nitric oxide scavenging and extravasation, high P50, and hyperoxygenation [10, 11]. Systemic and pulmonary vasoconstriction are considered a major concern following the administration of polymerized bovine Hb (PBH) to hypovolemic pigs, sheep, and humans [12]. Purity aside, multiple studies have demonstrated that the administration of large doses of concentrated (13 g/dL), low affinity (high P50) HBOCs results in systemic and pulmonary vasoconstriction (increased systemic and pulmonary pressures) and a reduction in functional capillary density (FCD; 13). Alternatively, exchange transfusion and hemorrhagic shock experiments examining the effects of lower concentrations (≤8 g/dL) of a low affinity (P50 ≥ 34 mm Hg) polymerized bovine Hb suggest that beneficial tissue perfusion pressure, FCD, and tPO_2_ can be maintained providing plasma Hb concentrations (Hb_p_) do not exceed 1.4 g/dL [13, 14].

We investigated the acute dose response characteristics of a low concentration (6.5 g/dL) of highly purified PBH solution (OC99; New A Innovation Ltd., Kowloon, Hong Kong) on systemic and pulmonary arterial blood pressures, tissue oxygenation, and the metabolic correlates of oxygen debt in an experimental model of controlled hemorrhagic shock in dogs. OC99 dose selection was based upon pilot experiments wherein the Hb_p_ concentration that prevented pulmonary hypertension (mean pressure > 25 mm Hg) in hemorrhaged dogs was identified [15]. We hypothesized that OC99 could be administered at doses that would improve tPO_2_ without producing pulmonary hypertension.

## 2. Materials and Methods

All procedures were reviewed and approved by the Institutional Animal Care and Use Committee at QTest Labs and complied with federal guidelines for the care and use of laboratory animals and were conducted according to the principles in the “Guide for the Care and Use of Laboratory Animals,” Institute of Laboratory Animals Resources, Eighth Edition (Washington, D.C.: The National Academies Press, 2011) and the Animal Welfare Act as amended.

Thirty-three young mature male Beagle dogs were acclimated to the animal facility during a seven-day quarantine period. All dogs were determined to be in good health based upon a physical examination, body temperature, heart and respiratory rate, general appearance, auscultation of the thoracic cavity, and palpation of the abdominal cavity. Dogs were individually housed in cages and exercised daily during the in-life phase of the study. The environmental conditions of the animal room were a light/dark cycle set to maintain approximately 12 hours of light and 12 hours of dark each day and a room temperature and relative humidity of 64 to 74°F and 30 to 60%, respectively. The room temperature and humidity were recorded hourly and monitored daily. The dogs were provided commercial dry dog food and water ad libitum.

### 2.1. Animal Preparation

Vascular catheters were placed in the cephalic and saphenous veins for administration of anesthetic drugs (cephalic vein) and treatments (saphenous vein), respectively. Dogs were administered butorphanol, 0.25 mg/kg IV, and anesthetized with propofol, 3.5 to 6 mg/kg IV, to facilitate orotracheal intubation. All dogs were mechanically ventilated with a volume-cycled ventilator (~12 breaths/min, tidal volume: 10 to 15 mL/kg) in order to maintain the arterial blood carbon dioxide (CO_2_) partial pressure (PaCO_2_) between 38 and 48 mm Hg. Anesthesia was initially maintained with 1.5% isoflurane in room air. Core body temperature ranged from 37 to 39°C and was maintained with temperature-controlled warm air blankets. The dogs were initially placed in right lateral recumbency, skin electrodes were attached, and a catheter introducer was placed in the left jugular vein through which a thermodilution catheter was introduced and positioned with the thermistor in the main pulmonary artery. The left carotid artery was isolated and a dual tipped pressure sensing catheter (Milar, Inc. Houston, TX) was positioned in the ascending aorta and left ventricle, respectively. These catheters permitted the determination of core body temperature (°C), pulmonary artery pressure (mm Hg), cardiac output (L/min) via thermodilution (Baxter, Deerfield, IL), and aortic pressure (mm Hg; EMKA Technologies, Falls Church, VA). The left or right femoral artery and vein were isolated and catheterized in order to permit hemorrhage and blood sampling, respectively. Once instrumented the dog was repositioned in dorsal recumbency for the remainder of the experiment. A 5 to 6 cm midline incision was made through the skin and linea alba and an intestinal loop of gut (jejunum) was exposed. A 20-gauge needle was used to establish a tract between the serosa and muscular layers of the intestine for probe placement and determination of intestinal mucosa tissue-oxygen tension (mm Hg) via optical fluorescence (OxyLite; Oxford Optronix, Abingdon, United Kingdom). The dogs were stabilized for approximately 30–45 minutes following instrumentation and administered 100 units/kg/h IV heparin in order to facilitate hemorrhage and blood sampling. Baseline data were recorded and blood samples obtained for determination of blood pH, gases (PaO_2_; PaCO_2_) and biochemical data (lactate; base excess). Each dog was then hemorrhaged to a mean arterial pressure ranging from 30 to 40 mm Hg by controlled bleeding (blood withdrawal or infusion). Hemorrhaged blood was collected into a heparinized collection bag and weighed. Arterial lactate and base excess and their ratio were monitored every 15 minutes during bleeding.

### 2.2. Study Design and Treatment Protocols

#### 2.2.1. Pilot Study

A pilot potency assay was performed in six anesthetized and hemorrhaged dogs weighing 10 ± 0.3 kg to determine the plasma hemoglobin concentration (g/dL) that produced a pulmonary artery pressure >25 mm Hg. We considered, a priori, a mean pulmonary artery pressure >25 mm Hg to be hypertensive [15]. Six dogs were prepared as described and administered placebo (2 dogs; Hextend [HEX], Hospira, Inc., Lake Forest, IL) or an intravenous infusion (10 mL/kg) of different doses of OC99 every 30 minutes for a total of 4 treatments (i.e., 40 mL/kg total). The concentration and rate of OC99 administration varied for each infusion: 0.325 g/kg/10 min. (1 dog); 0.1 g/kg/10 min. (1 dog); 0.05 g/kg/10 min. (1 dog); and 0.325 g/kg/30 min. (1 dog). All treatments were delivered as volume-matched (10 mL/kg) intravenous infusions (OC99 in HEX) to a total volume of 10 mL. Hemodynamic data, Hct (%) total hemoglobin (Hb, g/dL), Hb_p_ (g/dL), pH, PO_2_ (mm Hg), PCO_2_ (mm Hg), lactate (mmol/L), BE (mEq/L), venous blood oxygen saturation (SvO_2_: %), and intestinal mucosa tPO_2_ (mm Hg) were determined immediately before (baseline) and at 5 (except CO), 10, 20, and 30 minutes after each infusion. Additional data were obtained at 60 and 90 minutes after the last infusion.

#### 2.2.2. Dose-Response Study

A controlled, dose response study was performed in 24 dogs weighing 10.7 ± 0.2 kg. Six dogs were assigned to one of four treatment groups (6 dogs per group) using a twofold randomization process. Dogs were randomly selected from the colony of enrolled animals. The dogs were bled to a predetermined degree of oxygen debt (oxygen debt ≈ 110 mL/kg) that predicated a 50% mortality based upon metabolic surrogates (arterial lactate of 6 to 9 mM/L; base excess > −12 mM/L; 3, 5). Subsequently, each dog was randomly assigned to receive one of the four treatments. Randomization lists (dog and group) were generated using statistical software (SAS System for Windows, Version 9.1 or higher; SAS Institute Inc., 2004). All test treatments were administered (KD Scientific, Inc.; Holliston, MA) for 60 minutes through the catheter placed in the saphenous vein.

The test articles included OC99 and two non-oxygen carrying control solutions: Lactated Ringer's Solution (LRS; Abbott Laboratories, North Chicago, IL) and 6% HEX. OC99 is a highly purified and polymerized (bis[3,5-dibromosalicyl] fumurate) bovine Hb dissolved in Ringer's Acetate solution to a concentration of 6.5 ± 0.5 g/dL. OC99 contains greater than 85% tetramers and has a shelf life of 12 months at 25°C (Table 1). Six dogs each were administered (1) 20 mL/kg/h of HEX and 10 mL/kg/hr of LRS (HEX + CTRL/LRS), (2) 20 mL/kg/hr of HEX and 0.065 g/kg OC99 (HEX + LOW), (3) 20 mL/kg/h of HEX and 0.325 g/kg OC99 (HEX + MID), or (4) 20 mL/kg/h of HEX and 0.65 g/kg OC99 (HEX + HIGH). OC99 dosing was completed with a subsequent LRS infusion to a volume of 10 mL in HEX + LOW and HEX + MID. A total of 30 mL/kg fluid was administered over 60 minutes.

Hematological and biochemical data were determined from arterial and venous whole blood samples collected before hemorrhage, every 15 min during bleeding and at predetermined times for the duration of the 3-hour experiment. The blood samples were analyzed for HCT and Hb_p_, pH, PO_2_, PCO_2_, arterial lactate (La), base excess (BE), and SvO_2_. The COP was determined at 3 time points: baseline (before hemorrhage), the end of hemorrhage immediately before fluid resuscitation, and at the end of treatment. Hemodynamic parameters were recorded at baseline, immediately before fluid resuscitation, and every 20 minutes after the start of treatment. Data included an electrocardiogram (ECG), heart rate (HR, beats/min.), mean right-atrial (RAP, mm Hg) and pulmonary-artery (PAP, mm Hg) systolic (SAP), diastolic (DAP), and mean (MAP) arterial pressures (mm Hg), and left ventricular end systolic (LVESP, mm Hg) and end diastolic pressures (LVEDP, mm Hg).

### 2.3. Statistical Methods

The primary end-points of this study were changes in Hb_p_ concentration, tissue oxygenation tPO_2_ arterial lactate, base deficit, and SvO_2_. All statistical tests were performed using computer software (SAS System for Windows, Version 9 of the programs; SAS Institute Inc., 2004). Statistical analyses were performed separately for each parameter. All data are reported as the mean ± the standard error of the mean (SEM). The analysis of variance for repeated measures (ANOVA) included dose, time, and the interaction of dose and time as fixed effects, and the animal as a random effect. The model took into account the correlations in the postdosing averages that were present due to monitoring the same animals over multiple time points following a given dosing through the use of a covariance structure. If the *F*-value exceeded the critical value, post hoc (Holm-Sidak) tests were conducted within the ANOVA to test for significant differences among dose groups/time intervals. A Benjamini and Hochberg method was utilized to control for the false discovery rate for these tests across all time intervals. Bonferroni corrections were applied, when applicable, to the significance levels of multiple comparisons in order to control the overall error rate associated with each dose group comparisons at a given time interval to no more than 5%. The significant level was *P* < 0.05 and values below *P* < 0.1 level were considered as indication of a notable trend.

## 3. Results

### 3.1. Pilot Dose Determination Experiments

Controlled hemorrhage (51 ± 3 mL/kg) produced anticipated hemodynamic and blood chemical changes consistent with the development of hypovolemic shock and oxygen imbalance. Mean systemic and pulmonary arterial blood pressure significantly decreased (MAP: 110 ± 6 to 43 ± 2 mm Hg; PAP: 18 ± 1 to 9 ± 1 mm Hg). Cardiac output (CO: 1.4 ± 0.1 to 0.4 ± 0.0 L/min) and left ventricular end diastolic pressure (LVEDP: 10 ± 1 to 1 ± 1 mm Hg) significantly decreased associated with significant (*P* < 0.05) increases in heart rate (HR: 123 ± 5 to 195 ± 16 bpm). The tPO_2_ was significantly decreased (31 ± 8 to 2 ± 3 mm Hg) after hemorrhage. Systemic and pulmonary vascular resistance values significantly increased after hemorrhage (dyn·sec/cm^5^: SVR: 6042 ± 387 to 9056 ± 308; PVR: 491 ± 94 to 2180 ± 359). Blood arterial lactate significantly increased (La: 1.4 ± 0.1 to 4.5 ± 0.5 mM/L) while arterial base excess (BE: −7 ± 1 to −13 ± 1 mM/L) and venous oxygen saturation (SvO_2_: 80 ± 3 to 24 ± 4%,) decreased significantly.

**Figure 1:**
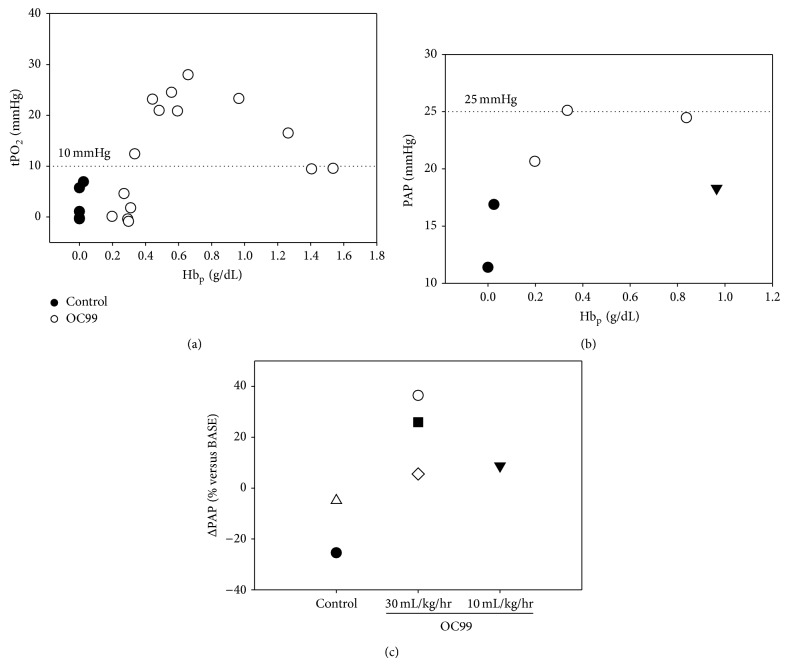
(a) OC99 plasma hemoglobin (Hb_p_) concentration versus tissue oxygen tension (tPO_2_). (b) OC99 Hb_p_ concentration versus mean pulmonary artery (PAP) pressure. (c) Change in PAP when 0.65 g/kg OC99 is administered at 30 ml/kg/hr or 10 ml/kg/hr; each symbol represents a different dog. Solid circles: Hextend without OC99; open circles: repeat doses of Hextend with increasing concentrations of OC99 administered over 10 minutes. Solid triangle: Hextend with OC99 administered over 30 minutes.

Infusion of OC99 produced dose and rate of administration dependent increases in Hb_p_. The Hb_p_ concentrations after the first and second infusions for the three doses of OC99 (0.325, 0.1, and 0.05 g/kg) administered over 10 minutes were 0.84, 0.34, and 0.20 g/dL and 1.26, 0.48, and 0.27 g/dL, respectively. The infusion of OC99 produced Hb_p_ concentration dependent increases in tPO_2_ in the intestinal mucosa. The greatest increases in tPO_2_ occurred at a Hb_p_ concentration averaging 0.84 ± 0.13 g/dL. The administration of HEX infusions containing OC99 produced Hb_p_ concentrations below 0.3 g/dL resulting in little or no change in PAP or tPO_2_ (tPO_2_ < 10 mm Hg; Figure 1). Infusion of HEX (0.0 g/kg) and the first dose (0.05 g/kg) of OC99 failed to attain this plasma concentration. The administration of repeat doses of OC99 resulting in Hb_p_ values greater than 0.3 g/dL increased pulmonary artery pressure; Hb_p_ concentration was an independent predictor of the mean pulmonary artery pressure (Figure 1). Pulmonary artery pressures exceeded 25 mm Hg when repeat dosages of 0.325 g/kg OC99 were administered over 10 minutes. We considered, a priori, a mean pulmonary artery pressure >25 mm Hg to be hypertensive [16]. The slower infusion of OC99 (0.325 g/kg over 30 min) prevented the increase in PAP. Based upon these results we hypothesized that the slow administration of OC99 to hemorrhaged dogs would increase tPO_2_ without producing pulmonary hypertension (i.e., mean PAP > 25 mm Hg) and when Hb_p_ concentration was less than 1.1 g/dL. Subsequent dose response experiments were designed to encompass OC99 Hb_p_ concentrations (0.3–1.1 gm/dL) that improved tPO_2_ without producing pulmonary hypertension.

## 4. Dose Response Experiments

Twenty-seven dogs were subjected to controlled hemorrhage. Two animals died during or immediately after hemorrhage and were not considered for analysis, while one dog did not meet entry criteria for lactate and base-deficit.

Data were collected and analyzed from twenty-four dogs. Baseline hemodynamic, blood chemistry, and tissue oxygenation values were considered to be within the normal range for dogs (Table 2). Controlled hemorrhage (644 ± 18 mL; 60 ± 2 mL/kg) produced the targeted metabolic end points (i.e., entry criteria LA 6 to 9 mM/L; BE > −12 mM/L) in 69 ± 4 min. Significant hemodynamic and metabolic changes consistent with the development of hypovolemic shock and oxygen imbalance were produced in all dogs (Table 2). Systemic and pulmonary arterial blood pressures (MAP: 40 ± 1 mm Hg; PAP: 20 ± 0.5 to 14 ± 0.4 mm Hg), cardiac output (CO: 0.4 ± 0.0 L/min), and left ventricular filling pressures (LVEDP: 2 ± 0.3 mm Hg) were significantly (*P* < 0.05) decreased and heart rate (210 ± 5 bpm) was significantly increased in all dogs. Systemic and pulmonary vascular resistance significantly increased after hemorrhage (Table 2). Oxygen extraction (ERO_2_) significantly increased. Intestinal tPO_2_ significantly decreased and arterial LA and BE were significantly increased (Table 2).

**Figure 2:**
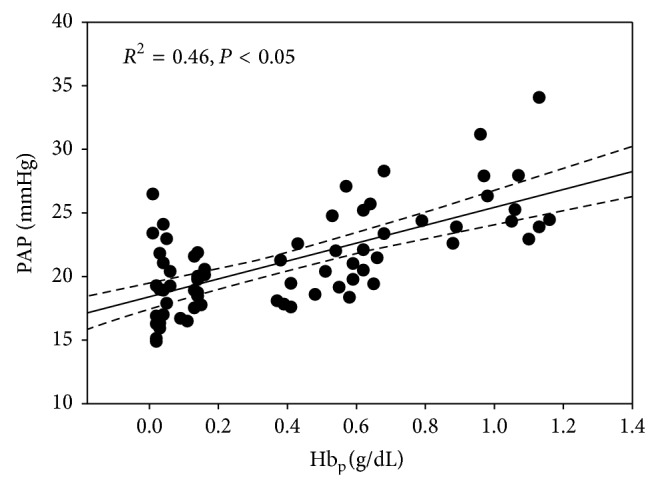
Effect of increasing plasma hemoglobin (Hb_p_) concentrations on mean pulmonary artery pressure (PAP) in hemorrhaged dogs.

Fluid resuscitation (30 mL/kg over 60 min) produced acute significant changes in the magnitude and sustainability of hemodynamic and blood chemical values in all fluid treatment groups. Larger doses of OC99 (0.325 g/kg; 0.65 g/kg) normalized or improved systemic pressures, CO and biochemical markers of anaerobic metabolism (LA, BE), and tPO_2_ (Table 3). Increasing Hb_p_ concentrations of OC99 increased PAP (Figure 2). The administration of 0.650 g/kg OC99 produced a significant increase in both SVR and PVR, and mean PAP at the end of dosing (Tables 3 and 4).

**Figure 3:**
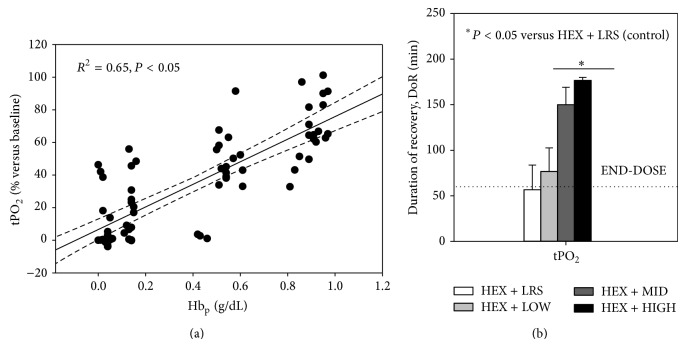
Effect of increasing plasma hemoglobin (Hb_p_) concentrations on tissue oxygen tension (tPO_2_) in hemorrhaged dogs. (a) Two-dimensional plot of Hb_p_ vs tPO_2_. (b) Effect of increasing Hb_p_ on the duration of tPO_2_ above 10 mm Hg.

There were no significant differences in posttreatment hematocrit concentration(s) among groups (Table 3). Increases in tPO_2_ were independent of Hct and COP and dependent upon OC99 Hb_p_ concentration (Table 3; Figure 3). Posttreatment tPO_2_ values > 10 mm Hg were maintained for a longer duration in dogs administered 0.325 and 0.65 g/kg OC99 (Table 5). Dogs administered 0.325 and 0.65 g/kg OC99 had significantly improved LA and BE values at the end of the study (i.e., at END) when compared to values after hemorrhage (Table 3).

## 5. Discussion

We determined the effects of increasing circulating concentrations of a highly purified PBH (OC99) on hemodynamics, metabolic correlates of oxygen debt (lactate, base deficit), and tissue oxygenation in a large animal model of controlled hemorrhagic shock. The administration of 0.325 g/kg of OC99 (plasma Hb ~0.6 g/dL) in conjunction with a colloid (Hextend) improved hemodynamics and tPO_2_ without producing significant increases in pulmonary artery blood pressure. The administration of 0.650 g/kg OC99 (plasma Hb ~1.0 g/dL) produced further increases in tPO_2_ and increased mean pulmonary arterial pressure to values greater than 25 mm Hg.

Multiple studies conducted in various experimental animal shock (anemia, controlled and uncontrolled hemorrhage) models have demonstrated the ability of various HBOC solutions to improve hemodynamics, blood oxygen content, oxygen delivery, tPO_2_, and survival [13, 16–21]. Whether or not the beneficial effects produced translate to clinical benefit however remains controversial based upon the potential for most HBOCs to produce oxidative stress, vasoconstriction, and methemoglobinemia [11]. Oxidation of cell-free Hb results in methemoglobin (heme-Fe^+2^) limiting the ability of Hb to carry oxygen and facilitating the production of tissue injury by free radicals [22]. Vasoconstriction is primarily attributed to the extravasation of cell-free Hb molecules through the endothelium with subsequent binding to nitric oxide produced by endothelial cells and to hyperoxygenation [22, 23]. Hyperoxygenation interferes with small vessel autoregulatory oxygen sensing mechanisms that normally trigger pulmonary arterial vasoconstriction and systemic arterial vasodilation when PaO_2_ decreases [11]. Additional factors that may contribute to vasoconstriction include the effects of cell-free Hb on endothelin release, adrenergic receptor activation, and the metabolism of arachidonic acid [22]. Systemic and pulmonary vasoconstriction may result in systemic or pulmonary hypertension, an increase in afterload leading to a decrease in cardiac output, myocardial energetic imbalance, ventilation-perfusion mismatch, a reduction in tissue perfusion, and heterogeneous changes in regional tissue perfusion. Furthermore, an increase in systemic pressure does not necessarily translate to increased perfusion of the microcirculation if arteriolar vasoconstriction is excessive or heterogeneous among tissue beds [14, 24].

Experimental studies in anesthetized dogs and swine that have been bled to a mean systemic arterial pressure of 50 mm Hg and conscious hemorrhaged and anemic hamsters implanted with window-chambers in their cheek pouches in order to visualize the microcirculation indicate that the administration of a 12-13 g/dL HBOC solution failed to return cardiac output and oxygen delivery to baseline values and reduced functional capillary density (the numbers/length of capillaries per unit area of tissue with visible RBC transit), respectively, despite an increase in blood oxygen content [13, 21, 25, 26]. Notably, a decrease in FCD has been directly linked to survival while vasoactive activity has been linked to NO scavenging, Hb_p_, and enhanced tissue oxygen tension [26]. Subsequent experiments, however, in severely hemorrhaged (≥50% blood volume) conscious hamsters, have demonstrated that administration of reduced concentrations of 4 to 8 g/dL HBOC maintained blood pressure without significant vasoconstriction and increased arteriolar oxygen supply and tPO_2_ [27]. These studies emphasize that the severity of blood loss is a determinant of the probability of a beneficial response to a HBOC (i.e., >blood loss produced better response) and the importance of the dose (mL/kg), concentration (g/dL), and rate of administration of the HBOC in determining Hb_p_ concentration and their subsequent efficacy and safety. This information combined with knowledge of the increased sensitivity of the pulmonary vasculature to the vasoconstrictive effects of HBOC solutions support the opinion that HBOC solutions should be considered as drugs and that their administration should be guided by the Hb_p_ concentration, pharmacokinetics, and the setting (anemia versus hemorrhage) in which they are administered [12, 28].

We utilized a validated hypovolemic hemorrhagic acute shock model designed to produce severe oxygen imbalance, resulting in the accumulation of oxygen debt resulting in a mortality >50% within 2 to 3 hours [5]. Heparin was administered to facilitate hemorrhage and blood sampling and may have interfered with inflammatory cell adhesion to the vascular endothelium thereby blunting the inflammatory response to hemorrhage [29, 30]. Our experiments were not designed to assess the effects of OC99 on the inflammatory cascade after shock, but to evaluate the effects of varying degrees of OC99 on hemodynamics and tissue oxygenation, variables that were unlikely to be affected by heparin administration. The administration of OC99 dose dependently normalized surrogates of oxygen debt (lactate and base deficit) and improved gut tPO_2_. Importantly, dogs administered 0.325 and 0.650 g/kg OC99 demonstrated increases in tPO_2_ to values previously demonstrated, in skeletal muscle and gut [31, 32], to be physiologically relevant (>10 mm Hg) and maintained these values for a significantly longer time than controls (0.0 g/dL). These effects became evident by the end of OC99 administration and were independent of both the intrinsic oxygen-carrying capability of the blood (i.e., Hct) and the resuscitation therapy administered (i.e., COP matched). The administration of 0.325 and 0.650 g/kg of OC99 exerted salutary effects on all variables associated with oxygen delivery, biochemical markers (LA and BE), and tPO_2_ leading to better recovery after hemorrhage compared to control dogs. Notably, the administration of 0.325 g/kg OC99 did not produce clinically relevant increases in PAP (i.e., >25 mm Hg) while 0.65 g/kg OC99 resulted in a pulmonary vasopressor effect by the end of the dosing period (26% increase from prehemorrhage values). Experimental studies in hemorrhaged swine and newborn lambs suggest that the pulmonary vasopressor effect is caused by cell free Hb_p_ and NO dysregulation and that breathing low levels (30–80 ppm) of NO or administration of various NO donors (nitroglycerin, sodium nitroprusside, and sodium nitrite) is palliative [33, 34]. Dissimilarities between systemic and pulmonary vasoconstrictive responses to cell-free Hb are attributed to phenotypic differences and a greater dependency of the muscularized pulmonary circulation to the vasoregulatory effects of NO [35].

Our studies indicate that the administration of 0.325 and 0.650 g/kg OC99 produced Hb_p_ concentrations ranging from approximately 0.4 g/dL and 1.1 g/dL, respectively, and that Hb_p_ values ≤0.8 g/dL do not produce a significant increase in pulmonary artery pressure. The data support and extend earlier studies investigating the systemic, pulmonary, and microcirculatory effects of HBOCs by identifying that Hb_p_ concentrations greater than 1.1 g/dL may cause systemic and pulmonary vasoconstriction limiting increases in tPO2 [14, 36]. Although this study was not designed to demonstrate improved survival two dogs in the low OC99 dose group (0.065 g/kg) died before the 180 min postdosing time point. No other premature deaths were observed and the hemodynamic and biochemical values recorded from these animals were similar to dogs in the control group suggesting that their death was likely attributable to the severity of the experimental procedures.

In conclusion, relatively low OC99 Hb_p_ concentrations augment tissue oxygen tension without producing adverse systemic or pulmonary vascular consequences. The administration of OC99 in conjunction with standard colloid-based fluid resuscitation produced dose-dependent increases in Hb_p_ concentration, systemic and pulmonary artery pressures, oxygen delivery, tPO_2_, and normalized clinical surrogates of tissue oxygen debt in a dog model of severe controlled hemorrhagic shock. These effects were independent of Hct and COP and suggest that OC99 can be administered as an adjunct to standard replacement fluid therapy to improve tissue oxygenation following hemorrhage. The data also suggest that pulmonary artery pressure is a more sensitive indicator of the response to cell-free Hb_p_ concentration than systemic pressure and should be monitored in order to avoid the potentially detrimental effects of vasoconstriction.

## Figures and Tables

**Table 1:** Comparison of composition of OC99 (Oxapex), HBOC-301 (Oxyglobin), and HBOC-201 (Hemopure).

	OC99 (Oxapex)	HBOC-301 (Oxyglobin)	HBOC-201 (Hemopure)
Hemoglobin concentration (g/dL)	6-7	12–14	12–14
Met-hemoglobin (%)	≤5%	<10	<10
*P*50 (mmHg)	39	40	40
pH	7.2–7.6	7.6–7.9	7.6–7.9
Endotoxin (EU/mL)	<0.05	<0.5	<0.5
Phospholipid (nmol/mL)	nd	<3	<3
Colloid osmotic pressure (mmHg)	19	42	25
Osmolarity (mOsm/kg)	250–350	300	300
Tetrameric hemoglobin (%)	>85	≤35	≤2
Average molecular weight (kDa)	65	200	250
Viscosity (centripoise at 37°C)	1.1	1.3	1.3
Half-life in humans/animals (hours)	16–55∗	18–43	19
Cross-linking agent	bis (3,5-dibromosalicyl) fumarate.	gluteraldehyde	gluteraldehyde
Storage conditions (°C)	<25 (without freezing)	2–30	2–30

^*^Dose dependent; nd: nondetectable.

**Table 2:** Representative systemic/left-ventricular hemodynamic, mechanical, and metabolic parameters measured before (i.e., at baseline) and after the induction of shock/oxygen imbalance via controlled hemorrhage in the isoflurane anesthetized closed-chest canine preparation.

Model characteristics (*n* = 24)		
Parameter (units)	Baseline	Hemorrhagic shock^5^
Hematocrit, PCV (%)^4^	41.9 (1.2)	40.5 (0.9)
Total Hb (g/dL)	14.3 (0.4)	13.8 (0.3)
Plasma Hb (g/dL)	0.06 (0.01)	0.07 (0.01)
MAP (mmHg)	105 (3)	40 (1)∗
SVR (dyne/cm/s^−5^)	4,192 (121)	7,053 (305)∗
PAP (mmHg)	20.4 (0.6)	14.6 (0.5)∗
PVR (dyne/cm/s^−5^)	471 (18)	2,584 (199)∗
HR (bpm)	131 (4)	212 (5)∗
LV-dP/dt_max_ (mmHg/s)^1^	+2,249 (85)	+1,214 (52)∗
LV-dP/dt_min_ (mmHg/s)^1^	−2,152 (82)	−822 (42)∗
Cardiac output (L/min)^2^	1.9 (0.1)	0.4 (0.0)∗
Oxygen extraction ratio (%)	12.8 (0.9)	67.6 (1.2)∗
PaO_2 _(mmHg)	82.5 (1.5)	86.4 (1.4)∗
PaCO_2 _(mmHg)	38.9 (0.6)	27.9 (0.9)∗
tPO_2_ (mmHg)^3^	32.2 (1.6)	2.7 (0.6)∗
pHa	7.30 (0.01)	7.19 (0.01)∗
BE (mM/L)^4^	−7.2 (0.4)	−17.5 (0.6)∗
LA (mM/L)^4^	2.2 (0.1)	7.2 (0.2)∗
SvO_2_ (%)	82.6 (0.9)	30.4 (1.1)∗

Data are mean values (standard error of the mean).

^1^Derived from left-ventricular pressure (LVP) signals.

^2^Measured/derived via thermodilution.

^3^Measured directly via optical fluorescence in the intestine.

^4^Measured/derived from arterial blood samples.

^5^The average volume of blood shed during the hemorrhagic shock period was 644 ± 18 mL.

^*^ *P* < 0.05 (statistically significant) versus baseline.

**(a):** 

Parameter (units)	HEX + CTRL				HEX + LOW				HEX + MID			
	Shock	End-dose	+1hr	End	Shock	End-dose	+1hr	End	Shock	End-dose	+1hr	End
PCV (%)	**39.5 (1.3)**	21.3 (1.7)^†^	21.3 (1.4)^†^	19.9 (1.4)^†^	**39.3 (1.8)**	20.3 (0.6)^†^	19.4 (0.9)^†^	19.9 (1.0)^†^	**42.5 (2.8)**	20.8 (0.9)^†^	20.3 (1.1)^†^	21.2 (1.3)^†^
Total Hb (g/dL)	**13.5 (0.4)**	7.3 (0.6)^†^	7.3 (0.5)^†^	6.8 (0.5)^†^	**13.5 (0.6)**	7.1 (0.2)^†^	6.7 (0.3)^†^	6.9 (0.3)^†^	**14.5 (0.9)**	7.7 (0.3)^†^	7.5 (0.4)^†^	7.7 (0.5)^†^
Plasma Hb (g/dL)	**0.06 (0.01)**	0.03 (0.01)^†^	0.03 (0.01)^†^	0.03 (0.01)^†^	**0.07 (0.02)**	0.15 (0.00)^∗†^	0.14 (0.01)^∗†^	0.14 (0.00)^∗†^	**0.07 (0.01)**	0.63 (0.01)^∗†^	0.59 (0.02)∗	0.53 (0.02)^∗†^

MAP (mmHg)	**41.7 (2.3)**	90.3 (6.7)^†^	84.7 (7.0)^†^	63.1 (7.3)^†^	**36.9 (1.2)**	96.1 (4.1)	79.3 (7.0)^†^	56.3 (5.1)^†^	**41.5 (0.9)**	114.3 (6.3)∗	107.2 (9.3)∗	89.3 (8.3)
PAP (mmHg)	**14.4 (0.6)**	20.3 (1.9)	17.3 (1.7)^†^	16.8 (1.7)^†^	**15.2 (1.4)**	21.7 (2.0)	17.0 (1.4)	16.0 (1.2)^†^	**14.7 (0.8)**	22.4 (1.5)	17.5 (0.9)	16.1 (1.2)^†^
HR (bpm)	**195.7 (10.0)**	187.6 (8.3)^†^	196.9 (9.6)^†^	209.6 (10.0)^†^	**213.1 (7.7)**	200.3 (6.7)^†^	203.4 (6.9)^†^	196.5 (4.1)^†^	**228.5 (6.3)**	204.6 (6.2)^†^	203.0 (8.2)^†^	201.5 (8.2)^†^
CO (L/min)	**0.4 (0.0)**	1.8 (0.1)	1.4 (0.1)	1.0 (0.1)^†^	**0.4 (0.1)**	1.9 (0.1)	1.3 (0.1)^†^	1.0 (0.1)^†^	**0.4 (0.0)**	2.0 (0.2)	1.5 (0.1)^†^	1.2 (0.1)^†^

O_2_ extraction (%)	**67.1 (3.4)**	21.3 (4.3)	36.2 (6.3)^†^	55.4 (8.0)^†^	**66.0 (2.5)**	27.1 (1.3)^†^	44.6 (4.6)^†^	57.8 (4.0)^†^	**70.3 (2.7)**	24.4 (2.6)^†^	32.8 (5.1)^†^	42.3 (5.8)^†^
PaO_2_ (mmHg)	**84.8 (2.6)**	81.5 (2.6)	80.9 (1.3)	83.0 (2.6)^†^	**91.8 (2.8)**	78.8 (1.8)	83.4 (3.6)^†^	86.6 (3.0)^†^	**86.0 (1.9)**	80.8 (3.1)	80.7 (3.0)	82.8 (4.2)^†^
PaCO_2_ (mmHg)	**29.2 (2.1)**	37.5 (1.3)	34.9 (1.1)	29.9 (1.6)	**27.6 (1.8)**	38.2 (0.7)	34.4 (1.5)	32.0 (1.2)	**26.7 (1.6)**	36.4 (0.5)	35.7 (1.0)	32.2 (1.5)
tPO_2_ (mmHg)	**1.1 (0.9)**	10.8 (4.4)^†^	8.4 (3.5)^†^	3.5 (2.8)^†^	**2.1 (1.1)**	19.3 (5.4)^†^	17.0 (4.9)^†^	5.9 (2.2)^†^	**2.8 (1.4)**	19.5 (2.7)^†^	19.3 (2.3)^∗†^	12.9 (3.0)^†^

pHa	**7.20 (0.02)**	7.28 (0.01)	7.33 (0.01)	7.34 (0.01)	**7.19 (0.04)**	7.29 (0.02)	7.36 (0.02)^†^	7.33 (0.03)	**7.17 (0.01)**	7.33 (0.02)^†^	7.37 (0.01)^†^	7.37 (0.03)^†^
BE (mM/L)	**−17.0 (0.5)**	−9.2 (0.6)	−7.3 (0.5)	−9.4 (1.3)	**−18.7 (1.3)**	−8.8 (1.1)	−6.2 (0.9)	−8.4 (1.5)	**−18.7 (0.9)**	−6.7 (1.2)	−4.8 (1.3)^†^	−6.3 (2.2)
LA (mM/L)	**7.0 (0.4)**	4.8 (0.6)^†^	3.8 (0.5)^†^	5.0 (1.0)^†^	**7.3 (0.4)**	4.9 (0.5)^†^	3.9 (0.6)^†^	5.3 (1.1)^†^	**7.4 (0.2)**	4.2 (0.4)^†^	2.9 (0.4)	3.4 (0.9)
SvO_2_ (%)	**30.7 (2.9)**	74.2 (4.2)^†^	60.6 (6.0)^†^	42.6 (7.6)^†^	**32.2 (2.2)**	68.7 (1.3)^†^	52.9 (4.4)^†^	40.5 (3.9)^†^	**27.8 (2.6)**	71.8 (2.5)^†^	64.1 (4.8)^†^	55.1 (5.4)^†^

**(b):** 

Parameter (units)	HEX + CTRL				HEX + HIGH			
	Shock	End-dose	+1hr	End	Shock	End-dose	+1hr	End
PCV (%)	**39.5 (1.3)**	21.3 (1.7)^†^	21.3 (1.4)^†^	19.9 (1.4)^†^	**40.7 (1.5)**	20.5 (1.5)^†^	18.8 (1.2)^†^	20.1 (1.2)^†^
Total Hb (g/dL)	**13.5 (0.4)**	7.3 (0.6)^†^	7.3 (0.5)^†^	6.8 (0.5)^†^	**14.0 (0.5)**	8.0 (0.5)^†^	7.4 (0.4)^†^	7.7 (0.4)^†^
Plasma Hb (g/dL)	**0.06 (0.01)**	0.03 (0.01)^†^	0.03 (0.01)^†^	0.03 (0.01)^†^	**0.07 (0.02)**	1.11 (0.02)^∗†^	1.02 (0.02)^∗†^	0.91 (0.01)^∗†^

MAP (mmHg)	**41.7 (2.3)**	90.3 (6.7)^†^	84.7 (7.0)^†^	63.1 (7.3)^†^	**40.0 (1.9)**	110.8 (4.9)∗	108.9 (5.5)∗	81.0 (7.0)^∗†^
PAP (mmHg)	**14.4 (0.6)**	20.3 (1.9)	17.3 (1.7)^†^	16.8 (1.7)^†^	**14.0 (1.2)**	26.3 (1.7)^∗†^	19.8 (1.7)	17.5 (1.3)^†^
HR (bpm)	**195.7 (10.0)**	187.6 (8.3)^†^	196.9 (9.6)^†^	209.6 (10.0)^†^	**211.7 (15.4)**	192.4 (6.8)^†^	197.1 (6.3)^†^	206.3 (7.2)^†^
CO (L/min)	**0.4 (0.0)**	1.8 (0.1)	1.4 (0.1)	1.0 (0.1)^†^	**0.5 (0.0)**	2.1 (0.2)	1.6 (0.1)^†^	1.2 (0.1)

O_2_ extraction (%)	**67.1 (3.4)**	21.3 (4.3)	36.2 (6.3)^†^	55.4 (8.0)^†^	**67.1 (1.2)**	25.5 (2.5)^†^	37.7 (3.0)^†^	53.3 (2.3)^†^
PaO_2_ (mmHg)	**84.8 (2.6)**	81.5 (2.6)	80.9 (1.3)	83.0 (2.6)^†^	**82.8 (2.7)**	75.8 (2.5)	74.6 (2.0)	78.3 (3.5)^†^
PaCO_2_ (mmHg)	**29.2 (2.1)**	37.5 (1.3)	34.9 (1.1)	29.9 (1.6)	**28.3 (2.2)**	38.8 (1.5)	37.1 (1.0)^†^	32.2 (1.2)
tPO_2_ (mmHg)	**1.1 (0.9)**	10.8 (4.4)^†^	8.4 (3.5)^†^	3.5 (2.8)^†^	**4.9 (1.5)**	26.0 (3.0)∗	24.7 (0.5)∗	20.3 (1.2)^∗†^

pHa	**7.11 (0.01)**	7.28 (0.01)	7.33 (0.01)	7.34 (0.01)	**7.20 (0.02)**	7.34 (0.02)	7.39 (0.02)^†^	7.41 (0.01)^†^
BE (mM/L)	**−17.0 (0.5)**	−9.2 (0.6)	−7.3 (0.5)	−9.4 (1.3)	**−16.8 (1.5)**	−5.0 (1.3)∗	−2.7 (1.2)^∗†^	−4.2 (1.1)^∗†^
LA (mM/L)	**7.0 (0.4)**	4.8 (0.6)^†^	3.8 (0.5)^†^	5.0 (1.0)^†^	**6.9 (0.3)**	4.5 (0.5)^†^	2.9 (0.3)	3.4 (0.3)
SvO_2_ (%)	**30.7 (2.9)**	74.2 (4.2)^†^	60.6 (6.0)^†^	42.6 (7.6)^†^	**30.8 (1.2)**	70.0 (2.3)^†^	59.0 (3.0)^†^	44.6 (2.1)^†^

Data are mean values (standard error of the mean).

^*^ *P* < 0.05 (adjusted) versus HEX + LRS; ^†^ *P* < 0.05 (adjusted) versus baseline (BASE), excluding “Shock” (not compared).

**Table 4:** Means (with standard errors) for the plasma colloid-osmotic-pressure (COP) as well as the systemic (SVR) and pulmonary (PVR) vascular resistances as measured/estimated at baseline (Base) and at the end of treatment (End-dose).

Parameter (units)/group	Baseline	End-dose	%Change
COP (mmHg)			
HEX + CTRL	16.5 (0.5)	14.6 (0.4)	−11.1 (2.2)
HEX + LOW	16.3 (0.9)	14.6 (0.6)	−9.6 (4.5)
HEX + MID	16.4 (1.1)	16.6 (1.1)	1.7 (5.4)
HEX + HIGH	15.7 (0.9)	17.4 (0.7)	11.2 (3.3)
SVR (dyne/cm/s^−5^)			
HEX + CTRL	4,560 (171)	3,743 (236)	−17.9 (4.6)
HEX + LOW	4,201 (378)	3,975 (417)	−3.3 (8.9)
HEX + MID	4,160 (110)	4,415 (270)	6.3 (6.6)
HEX + HIGH	3,845 (187)	4,293 (205)	13.4 (8.8)∗
PVR (dyne/cm/s^−5^)			
HEX + CTRL	485 (51)	647 (75)	35.0 (9.5)
HEX + LOW	507 (47)	734 (83)	45.3 (12.6)
HEX + MID	440 (9)	676 (62)	53.4 (13.5)
HEX + HIGH	452 (17)	881 (70)∗	96.2 (16.2)∗

Data are mean values (standard error of the mean).

^*^ *P* < 0.05 (adjusted) versus HEX + LRS.

**Table 5:** Indices indicative of both hemodynamic (systolic blood pressure, SBP) and oxygenation (tissue oxygen tension, TO_2_) recovery^1^ following the administration of either the placebo (LRS) or the test article (OC99): time to recovery (TtR) and duration of recovery (DoR).

Group^†,‡^	tPO_2_ (>10 mmHg)			SBP (>90 mmHg)		
	TtR (min)	DoR (min)	%^2^	TtR (min)	DoR (min)	%^2^
HEX + CTRL	50.0 (19.1)	56.7 (27.0)	2/6	26.7 (4.2)	136.7 (15.8)	2/6
HEX + LOW	35.0 (9.6)	76.7 (26.0)	4/6	23.3 (3.3)	150.0 (13.4)	6/6
HEX + MID	33.3 (6.7)	150.0 (19.1)∗	6/6	20.0 (0.0)	170.0 (10.0)	6/6
HEX + HIGH	23.3 (3.3)	176.7 (3.3)∗	6/6	20.0 (0.0)	180.0 (0.0)∗	6/6
P-value	*0.8415 *	***0.0005***		*0.4402 *	***0.0178***	

Data are mean values (standard error of the mean).

^1^For the purposes of this study, “oxygenation” and “hemodynamic” recovery were defined as the time point(s) when the tissue (intestinal) oxygen tension (TO_2_) and the systemic (systolic) blood pressure (SBP) (resp.) reached values associated with sustainable normal physiological function (TO_2_ > 10 mmHg, SBP > 90 mmHg).

^2^The ratio of the number of animals that reached the set threshold level. For the TO_2_ parameter, there were 2 animals from the HEX + LRS group and 2 animals from the HEX + LOW group that did not reach the set threshold level.

^†^Treatments delivered via continuous IV infusion over 60 min at 10 mL/kg/hr (combined) in the setting of fluid-resuscitation (20 mL/kg at 20 mL/kg/hr) with either Hextend (HEX+) or LRS (LRS+); end of dosing (DOSE_END_) is time zero (*t *= 0 hr), while END is the mean value readings taken 2 hours after dosing (140, 160, and 180 min).

^‡^LRS (*n* = 6): 10 mL/kg of LRS; LOW (*n* = 6): 1 mL/kg of OC99 + 9 mL/kg of LRS; MID (*n* = 6): 5 mL/kg of OC99 + 5 mL/kg of LRS; HIGH (*n* = 6): 10 mL/kg of OC99.

^*^ *P* < 0.05 (adjusted) versus HEX + LRS.
